# New triazole-based coordination complexes as antitumor agents against triple negative breast cancer MDA-MB-468 cell line†

**DOI:** 10.1039/d3ra07714d

**Published:** 2023-12-12

**Authors:** Youssef Draoui, Smaail Radi, Yousra Bahjou, Abderrazak Idir, Amal El Mahdaoui, Abdelmajid Zyad, Haralampos N. Miras, Marilena Ferbinteanu, Aurelian Rotaru, Yann Garcia

**Affiliations:** a LCAE, Department of Chemistry, Faculty of Science, University Mohamed I P.O. Box 524 Oujda 60 000 Morocco s.radi@ump.ac.ma; b Institute of Condensed Matter and Nanosciences, Molecular Chemistry, Materials and Catalysis (IMCN/MOST), Université catholique de Louvain PlaceL. Pasteur 1, 1348 Louvain-la-Neuve Belgium yann.garcia@uclouvain.be; c Team of Experimental Oncology and Natural Substances, Cellular and Molecular Immuno Pharmacology, Faculty of Science and Technology, Sultan Moulay Slimane University Beni-Mellal Morocco; d School of Chemistry, Joseph Black Building, University of Glasgow Glasgow G12 8QQ UK; e Inorganic Chemistry Department, Faculty of Chemistry, University of Bucharest Panduri Road, No. 90 Bucharest 050663 Romania; f Department of Electrical Engineering and Computer Science & Research Center MANSiD, “Stefan cel Mare” University University Street, No. 13, Suceava 720229 Romania

## Abstract

The present work describes the synthesis of a new triazole based ligand 3-(3,5-dimethyl-1*H*-pyrazol-1-yl)-1-methyl-1*H*-1,2,4-triazole (LM) and demonstration of its coordination diversity giving rise to a family of seven new coordination complexes, namely: [Ni(LM)_3_](ClO_4_)_2_·C_2_H_6_OS (5), [Co_2_(LM)_6_](ClO_4_)_4_·(C_2_H_5_)O (6), [Cd(LM)_2_Cl_2_] (7), [Cu(LM)_2_NO_3_]NO_3_ (8), [Fe(LM)_3_](BF_4_)_2_ (9), [Zn(LM)_3_](BF_4_)_2_ (10) and [Zn(LM)_2_NO_3_]NO_3_ (11), whose crystal structure was determined by single-crystal X-ray diffraction. Cytotoxic activity was evaluated against the MDA-MB-468 cancer cell line, which serves as a model for triple-negative breast cancer, and compared to the precursor molecule (L), as well as their coordination complexes (H_3_O){[NiL_3_](ClO_4_)_3_} (1), [CoL_3_](ClO_4_)_2_·2H_2_O (2), [CdL_2_Cl_2_] (3) and [CuL_3_](NO_3_)_2_ (4), for which the crystal structure was earlier determined. Notably, cadmium complexes 3 and 7 exhibit remarkable cytotoxicity and demonstrated a high selectivity index towards cancer cells when compared to peripheral blood mononuclear cells. Such activity highlights their potential function as anticancer agents.

## Abstract 2

The synthesis of a new family of seven new coordination complexes, with remarkable cytotoxicity and high selectivity index towards cancer cells when compared to peripheral blood mononuclear cells (PBMCs) is reported.

## Graphical abstract

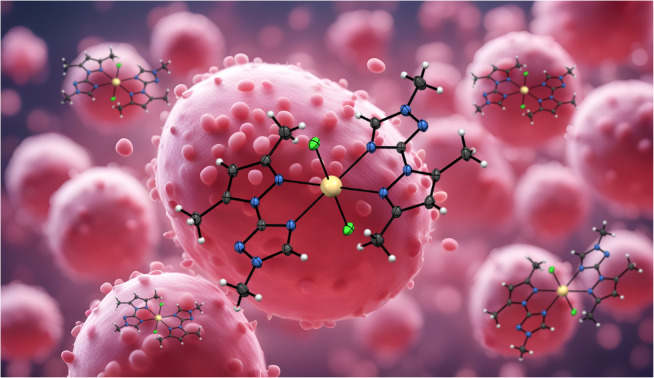

## 1. Introduction

Breast cancer represents a significant worldwide health concern, affecting millions of women each year.^1,2^ Triple-negative breast cancer (TNBC) stands out as a particularly aggressive and invasive type of cancer, and is considered to be the most difficult one to be treated effectively.^3^ It is characterized by the absence of estrogen receptor (ER), progesterone receptor (PR), and human epidermal growth factor receptor 2 (HER2) expression.^4^ According to Cancer Treatment Centers of America (CTCA), TNBC accounts for approximately 10–15% of all breast cancer cases, presenting unique clinical and biological features that contribute to its aggressive nature and very limited treatment options. Despite substantial progress in understanding and treating breast cancer, its heterogeneity poses challenges in managing effectively the disease.^5–7^

Notwithstanding the development of intense therapeutic techniques, chemotherapy medications remain an important tool in the fight against breast cancer.^8,9^ They are also regarded as the cornerstones of cancer therapeutic research.^10^ Current TNBC treatment techniques are still inadequate, and poly-chemotherapy is the usual treatment for early TNBC in order to assess tumor sensitivity.^5^ Acquired resistance, significant general systemic toxicity, and metastasis are the key barriers to successful chemotherapy.^11–13^ As a result, any advancement of new or additional chemotherapeutic solutions involves a highly challenging underlying chemistry. Thus, the development of innovative, alternative, and effective anticancer medicines for treating systemic relapse in breast cancer therapy remains a top priority.^14–16^

Coordination complexes can exhibit enhanced anticancer activity compared to individual ligands or metal ions alone. In fact, coordination process can modify chemical and physical properties of the ligands, leading to improved stability, solubility, and bioavailability.^17–20^ This enhanced stability allows coordination complexes to effectively interact with biological targets, such as cancer cells, and exert their anticancer effects. Furthermore, the introduction of metal ions into coordination complexes can introduce additional mechanisms of action that are not present in the ligands alone. Metal ions can interact with cellular components, such as DNA or proteins, leading to specific biological effects. These interactions can disrupt cellular processes, induce apoptosis, inhibit cell proliferation, or interfere with signaling pathways crucial for cancer cell survival and growth.

Much attention has been drawn to triazole-based compounds due to their versatility and their potential as anticancer pharmaceuticals throughout the last decade.^21–23^ A number of new chemicals containing a 1,2,4-triazole scaffold were developed and tested for anticancer activity against a panel of cancer cell lines.^24^ Furthermore, some highly promising outcomes were achieved in the case of 1,2,4-triazole-3-thiol derivatives against human melanoma IGR39, human triple-negative breast cancer (MDA-MB-231) and pancreatic carcinoma (Panc-1) cell lines. Their potential selectivity towards the same cells was also investigated.^25^ Interestingly, a large number of triazole based molecules demonstrated their efficiency against TNBC.^26^ Among those, Azeliragon triazole analogues have recently been identified for their excellent activities.^27^ Due to the triazole's high coordination affinity towards several transition metals, it was envisaged that the systematic investigation of relevant coordination complexes may open up new avenues and even more possibilities for the design and development of enhanced and more reliable anticancer agents.^28–31^

**Scheme 1:**
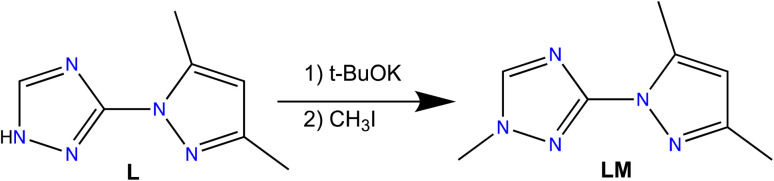
Synthetic route of LM.

**Scheme 2:**
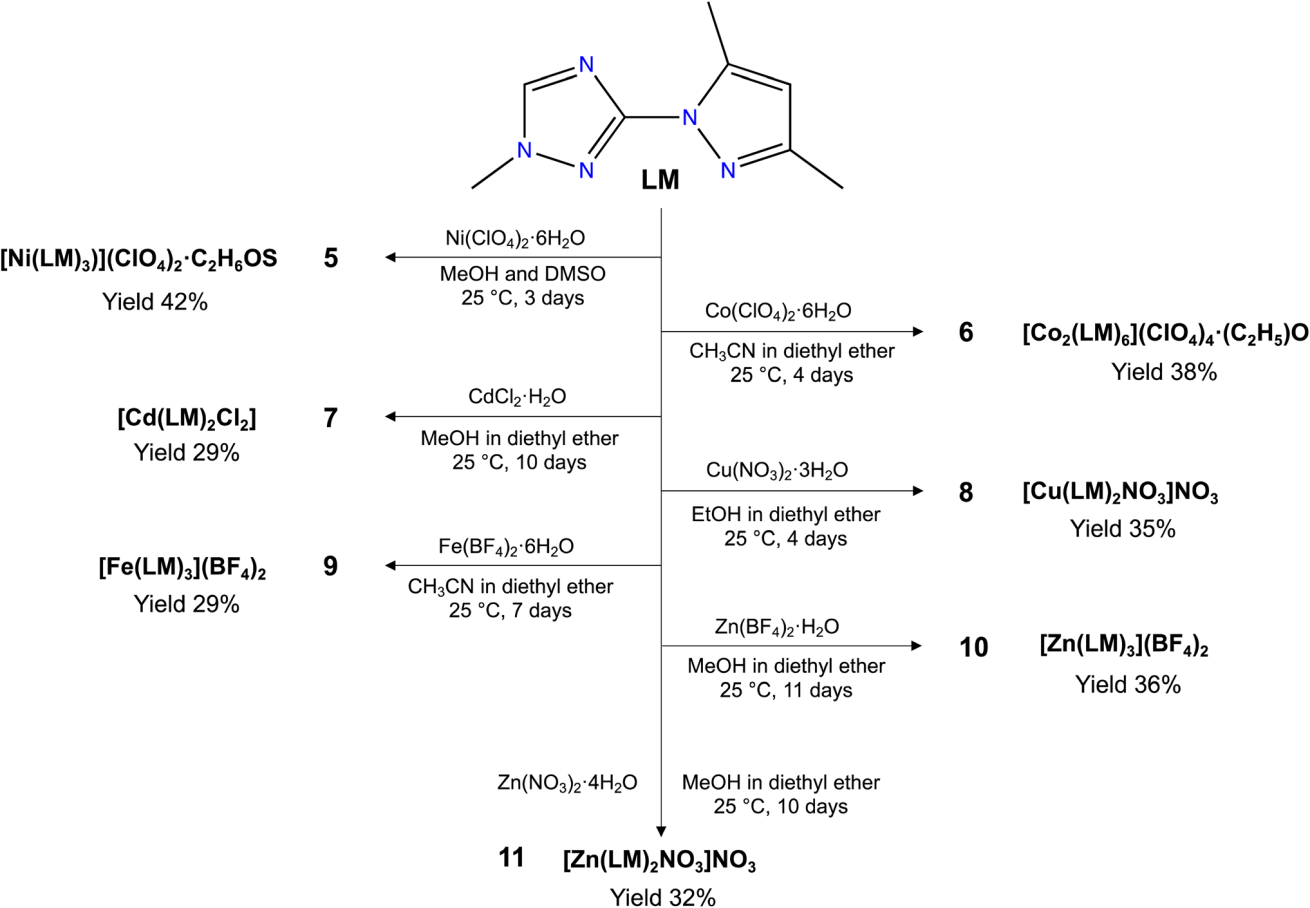
Synthesis of coordination complexes 5–11.

Herein, we report the syntheses and characterization a new triazole based ligand 3-(3,5-dimethyl-1*H*-pyrazol-1-yl)-1-methyl-1*H*-1,2,4-triazole (LM) (Scheme 1), that was characterized by different spectroscopic techniques, including ^1^H NMR, ^13^C NMR, FT-IR and UV-vis spectroscopies, and high-resolution mass spectrometry. The reaction of LM with different metal salt led to the formation of seven new coordination complexes, namely, [Ni(LM)_3_](ClO_4_)_2_·C_2_H_6_OS (5), [Co_2_(LM)_6_](ClO_4_)_4_·(C_2_H_5_)O (6), [Cd(LM)_2_Cl_2_] (7), [Cu(LM)_2_NO_3_]NO_3_ (8), [Fe(LM)_3_](BF_4_)_2_ (9), [Zn(LM)_3_](BF_4_)_2_ (10) and [Zn(LM)_2_NO_3_]NO_3_ (11) (Scheme 2). The structural features of these coordination complexes were investigated using single-crystal X-ray diffraction. Moreover, these complexes were evaluated against the MDA-MB-468 cancer cell line, which was used as a model for the triple-negative breast cancer. The precursor L and its coordination complexes (H_3_O){[NiL_3_](ClO_4_)_3_} (1), [CoL_3_](ClO_4_)_2_·2H_2_O (2), [CdL_2_Cl_2_] (3) and [CuL_3_](NO_3_)_2_ (4) that we previously reported for their antibacterial and antifungal properties,^32^ were also investigated and discussed for comparison.

## 2. Experimental section

### 2.1. Materiel and instrumentation

All solvents and chemicals, obtained from usual commercial sources, were of analytical grade and used without further purification. ^1^H and ^13^C NMR spectra were obtained using a Bruker AC 300 spectrometer. High resolution mass spectrometry HRMS data were obtained with a Q Exactive Thermofisher Scientific ion trap spectrometer by using ESI ionization. FT-IR spectra were recorded on KBr pellets using a PerkinElmer 1310 spectrometer. UV-visible spectra were recorded using a Shimadzu 3600 plus spectrometer equipped with Harrick praying mantis modulus which allows direct analysis of powders in reflectance mode. Elemental analysis was performed with a vario EL microanalyzer (C, H, N and O). Magnetic susceptibilities were measured on a Quantum design MPMS-5s SQUID magnetometer. Magnetic data were corrected for the sample holder and diamagnetic contributions. A polycrystalline sample of 9 was quickly loaded into a gelatine capsule and immediately inserted within the SQUID cavity to avoid any air oxidation. Mössbauer spectra were recorded in transmission geometry with a constant acceleration mode conventional spectrometer equipped with a 50 mCi ^57^Co(Rh) source and a Reuter Stokes proportional counter. The powdered sample was sealed in plastic sample holder and a spectrum was recorded at 298 K. The spectrum was fitted using Recoil 1.05 Mössbauer Analysis software.^33^ Isomer shift values are given with respect to α-Fe at room temperature.

### 2.2. X-ray crystallography

Suitable single crystals were selected and mounted onto a rubber loop using Fomblin oil. Crystal data for 5, 6, 8, 9 and 10 have been collected on a MAR345 Image plate detector using a monochromated (montel optics) microfocus Mo Kα radiation (*λ* = 0.71073 Å) source (Incoatec IμS). Data integration and reduction was performed using the CrysAlisPRO crystallographic software package and the implemented absorption correction was used.^34^

Crystal data for 7 have been collected on a Rigaku R-AXIS RAPID II diffractometer using graphite monochromated Mo Kα radiation (*λ* = 0.71075 Å) and the *ω*–*ϕ* scan technique. All structures were solved employing dual space direct methods (SHELXT),^35^ and refined by SHELXL2018/3.^36^ Nonhydrogen atoms were refined anisotropically with hydrogen placed in calculated positions and refined in riding mode.

Single-crystal X-ray diffraction data of 11 were recorded on a Bruker Apex CCD diffractometer (*λ* (Mo Kα) = 0.71073 Å) at 150 K equipped with a graphite monochromator. Structure solution and refinement were carried out with SHELXS-97,^37^ and SHELXL-97,^38^ using the WinGX software package.^39^ Data collection and reduction were performed using the Apex2 software package. Corrections for incident and diffracted beam absorption effects were applied using empirical absorption corrections.^40^ All atoms and most of carbon atoms were refined anisotropically. Solvent molecule sites were found and included in the refinement of the structures.

Table S1† provides the crystallographic data, data collection and refinement details for the compounds. Table S2 (ESI†) contains a summary of selected bond lengths [Å] and angles [°].

### 2.3. Synthesis section

L and 1–4 were prepared as previously reported.^32^

#### 2.3.1 3-(3,5-Dimethyl-1*H*-pyrazol-1-yl)-1-methyl-1*H*-1,2,4-triazole (LM)

To a solution of THF (80 mL) containing L, 3-(3,5-dimethyl-1*H*-pyrazol-1-yl)-1*H*-1,2,4-triazole (2 g, 0.0122 mol), *t*-BuOK (1.65 g, 0.0147 mol) was added to the mixture, and the reaction was refluxed for 2 h in a closed vessel. The reaction mixture then cooled using an ice bath, followed by the dropwise addition of iodomethane solution (1.91 g, 0.0134 mol) in THF (30 mL). The resultant mixture was refluxed again for 24 h, filtered and concentrated to dryness. Diethyl ether (15 mL) was added to the resulting oily product and the solution was chilled for two days at 0 °C leading to the isolation of colorless crystals of the product LM. Mp: 68 °C. Yield 81%, FT-IR: 3131(w), 2965(w), 1557(s), 1501(m), 1443(m) 1095(s), 629(w). ^1^H-NMR (DMSO) *δ* ppm: 2.16 (s, 3H, C*H*_3_–C=N), 2.36 (s, 3H, C*H*_3_–C–N), 3.89 (s, 3H, C*H*_3_–N), 6.05 (s, 1H, C=C*H*–C), 8.52 (s, 1H, N–C*H* = N). ^13^C-NMR (DMSO) *δ* ppm: 12.75 (s, 1C, *C*H_3_–C–N), 13.86 (s, 1C, *C*H_3_–C=N), 36.89 (s, 1C, *C*H_3_–N), 107.89 (s, 1C, C=*C*H–C), 141.34 (s, 1C, N–*C*=CH), 145.46(s, 1C, N–*C*H = N), 149.43(s, 1C, N=*C*–CH), 157.60(s, 1C, N–*C*–N). ESI-MS: *m*/*z* = 178.1087 (C_8_H_12_N_5_).

#### 2.3.2 [Ni(LM)_3_](ClO_4_)_2_·C_2_H_6_OS (5)

Ni(ClO_4_)_2_·6H_2_O (36.5 mg, 0.1 mmol, 1 equiv.) was dissolved in methanol (3 mL) and added to a solution of LM (53.1 mg, 0.3 mmol, 3 equiv.) dissolved in methanol (3 mL). 5 drops of DMSO were added to the reaction mixture and stirred for 10 min at 60 °C. The resulting clear reaction mixture was left at room temperature for slow evaporation. Violet single crystals were obtained after 3 days. Yield 42%. FT-IR: 3135(w), 2960(w), 1592(s), 1562(m), 1491(m), 1095(s), 619(w). ESI-MS: *m*/*z* = 688.1871 ([C_24_H_33_O_4_N_15_ClNi]).

#### 2.3.3 [Co_2_(LM)_6_](ClO_4_)_4_·(C_2_H_5_)O (6)

LM (53.1 mg, 0.3 mmol, 3 equiv.) was dissolved in acetonitrile (3 mL). Co(ClO_4_)_2_·6H_2_O (36.5 mg, 0.1 mmol, 1 equiv.) was also dissolved in acetonitrile (3 mL) and subsequently added to the solution of LM, and stirred for 10 min at room temperature. Orange single crystals were obtained after 4 days *via* vapour diffusion of diethyl ether (10 mL) at room temperature. Yield 38%. FT-IR: 3133(w), 2959(w), 1587(m), 1562(m), 1488(m), 1088(s), 618(w). ESI-MS: *m*/*z* = 295.1181 ([C_24_H_33_N_15_Co]).

#### 2.3.4 [Cd(LM)_2_Cl_2_] (7)

LM (35.4 mg, 0.2 mmol, 2 equiv.) was dissolved in methanol (3 mL). CdCl_2_·H_2_O (20.1 mg, 0.1 mmol, 1 equiv.) was also dissolved in methanol (3 mL) and five drops of water, and then added to the LM solution, and stirred for 10 min at room temperature. Colourless single crystals were obtained after 10 days *via* vapour diffusion of diethyl ether (10 mL) at room temperature. Yield 29%. FT-IR: 3103(w), 2992(w), 1596(s), 1551(m), 1471(m), 1103(m), 623(w). ESI-MS: *m*/*z* = 230.0541 ([C_16_H_22_N_10_Cd]).

#### 2.3.5 [Cu(LM)_2_NO_3_]NO_3_ (8)

LM (35.4 mg, 0.2 mmol, 2 equiv.) was dissolved in ethanol (3 mL). Cu(NO_3_)_2_·3H_2_O (24.1 mg, 0.1 mmol, 1 equiv.) was also dissolved in ethanol (3 mL) and added subsequently to the LM solution, and stirred for 10 min at room temperature. Green single crystals were obtained after 4 days *via* vapour diffusion of diethyl ether (10 mL) at room temperature. Yield 35%. FT-IR: 3125(w), 2988(w), 1601(s), 1562(m), 1466(m), 1101(m), 622(w). ESI-MS: *m*/*z* = 479.1197 ([C_16_H_22_O_3_N_11_Cu]).

#### 2.3.6 [Fe(LM)_3_](BF_4_)_2_ (9)

LM (53.1 mg, 0.3 mmol, 3 equiv.) was dissolved in acetonitrile (3 mL). Fe(BF_4_)_2_·6H_2_O (33.7 mg, 0.1 mmol, 1 equiv.) was also dissolved in acetonitrile (3 mL) in the presence of ascorbic acid (5 mg), and added to the LM solution, and stirred for 10 min at room temperature. Yellow colored single crystals were obtained after 7 days *via* vapour diffusion of diethyl ether (10 mL) at room temperature. Yield 29%. FT-IR: 3135(w), 2958(w), 1588(s), 1561(m), 1453(m), 1090(s), 623(w). Anal. Calcd (%) for C_24_H_33_FeN_15_, 2(BF_4_): C, 37.88; H, 4.37; N, 27.61. Found: C, 37.97; H, 4.35; N, 27.52.

#### 2.3.7 [Zn(LM)_3_](BF_4_)_2_ (10)

LM (53.1 mg, 0.3 mmol, 3 equiv.) was dissolved in methanol (3 mL). Zn(BF_4_)_2_·H_2_O (25.7 mg, 0.1 mmol, 1 equiv.) was also dissolved in methanol (3 mL) with five drops of water, and then added to the LM solution, and stirred for 10 min at room temperature. Colourless single crystals were obtained after 11 days *via* vapour diffusion of diethyl ether (10 mL) at room temperature. Yield 36%. FT-IR 3130(w), 2955(w), 1593(s), 1560(m), 1444(m), 620(w). ESI-MS: *m*/*z* = 297.6160 ([C_24_H_33_N_15_Zn]).

#### 2.3.8 [Zn(M)_2_NO_3_]NO_3_ (11)

LM (35.4 mg, 0.2 mmol, 2 equiv.) was dissolved in methanol (3 mL). Zn(NO_3_)_2_·4H_2_O (26.1 mg, 0.1 mmol, 1 equiv.) was also dissolved in methanol (3 mL) and added to the LM solution, and stirred for 10 min at room temperature. Colourless single crystals were obtained after 10 days *via* vapour diffusion of diethyl ether (10 mL) at room temperature. Yield 32%. FT-IR: 3135(w), 2995(w), 1595(s), 1521(m), 1454(m), 1104(m), 624(w). Anal. Calcd (%) for C_16_H_22_N_11_O_3_Zn, NO_3_: C, 35.34; H, 4.08; N, 30.91; O, 17.65. Found: C, 35.47; H, 4.03; N, 30.85; O, 17.69.

### 2.4. Anticancer activities

#### 2.4.1 Study of cytotoxic activity on cancer cells

The *in vitro* antitumor activity of L and LM, along with their metal complexes 1–4 and 5–11 respectively, was assessed against the MDA-MB-468 breast cancer cell line, following the [3-(4,5-dimethylthiazol-2-yl)-2,5-diphenyltetrazolium bromide] (MTT) assay as described in our previous study.^41^ Briefly, cells were seeded in 96-well microtiter plates at a density of 10^4^ cells per well. After overnight incubation, cells were exposed to different concentrations (0–400 μM) of each compound in 100 μL of culture medium. The plates were then incubated. Negative control (DMSO) and positive control (paclitaxel) were included, ensuring the final concentration of DMSO remained below 0.2%. After 48 h, 20 μL of MTT solution (5 mg mL^−1^) were added to each well, followed by a 4 h incubation. Subsequently, 150 μL of culture medium were replaced with 150 μL of isopropanol-HCl to dissolve the formazan crystals. All incubations were carried out in a humidified atmosphere at 37 °C and 5% CO_2_. The optical density was measured at *λ* = 540 nm, and cell viability was determined by calculating the percentage of absorbance of treated cells relative to untreated cells.

#### 2.4.2 Study of cytotoxic activity on non-cancer cells

Blood samples from healthy volunteer donors were used after approval by the Biomedical Research Ethics Committee. Peripheral blood mononuclear cells (PBMCs) were isolated using a density gradient method following the manufacturer's instructions (Capricorn Scientific). The PBMCs were then seeded into 96-well microtiter plates at a density of 2 × 10^4^ cells per well. The cytotoxic effect was evaluated on PBMCs under the same conditions and concentrations as previously described for tumor cells using the MTT assay.

#### 2.4.3 Statistical analysis

Data were first exported as an Excel file for initial analysis. Subsequently, viability values obtained were transferred to GraphPad Prism 8 for further analysis. A one-way ANOVA with Tukey's multiple comparisons test was conducted as a post hoc test. The data is presented as means ± SD from three independent experiments. Statistical significance was determined at a *p*-value of less than 0.05 in all cases.

## 3. Result and discussion

### 3.1. Synthesis of LM and its coordination complexes (5–11)

Considering our previously reported bioactive coordination complexes (1–4) using ligand L,^32^ we have decided to enhance the potential of this ligand by a straightforward alkylation using iodomethane. The synthesis procedure we documented is centred on utilizing *t*-BuOK and THF instead of other options for the base and solvent. This choice was made because it enabled the extraction of exceptionally pure LM without the need for additional purification, resulting in a higher yield.

Our aim was to explore how the choice of metal and counter anions impacts the formation and structural characteristics of our coordination complexes, while also evaluating their potential as anticancer agents. To achieve this, we employed various ligand/metal ratios, tested diverse metal salts with varying counter anions, and employed different crystallization methods/solvents. Among the compounds we examined, 5–11 emerged as the most promising and yielded high-quality single crystals suitable for X-ray analysis.

### 3.2. FT-IR and UV-visible spectroscopies

**Fig. 1:**
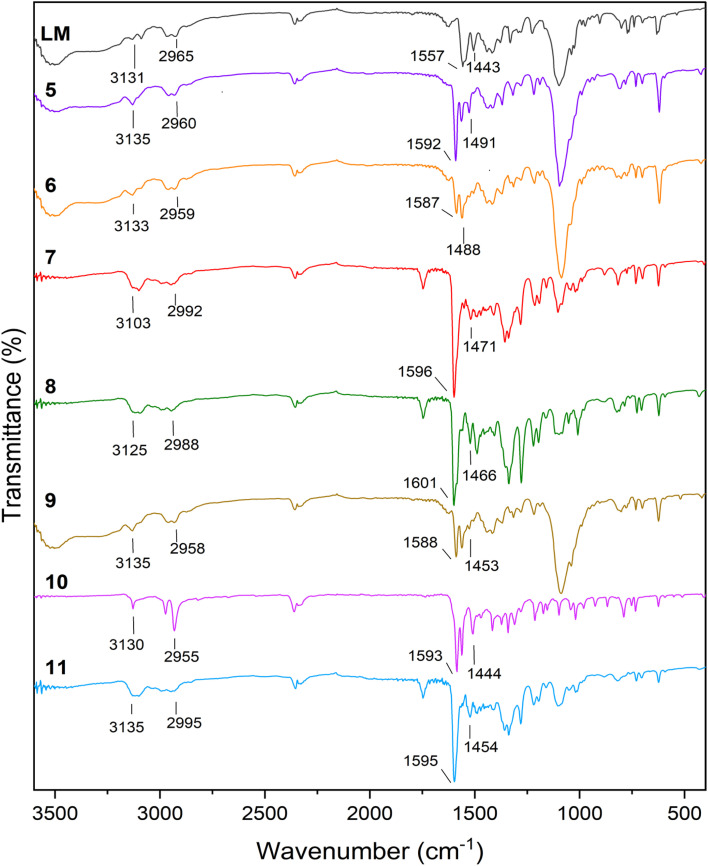
FT-IR spectra of LM and complexes 5–11.

Fig. 1 illustrates the FT-IR comparison plot of the ligand LM, and its relative coordination complexes. LM exhibits characteristic peaks located at 3131 and 3009 cm^−1^ both of which represent the N–H stretching vibration of triazole and pyrazole, respectively. Furthermore, due to the C–H aromatic vibration, a weak peak can be observed at 2965 cm^−1^. Additionally, the peaks centred at 1557 and 1443 cm^−1^ are assigned to the –N=N and C=C aromatic stretching vibrations. It is undoubtable that most of the representative ligand-based vibration peaks were shifted upon coordination with the transition metals. The biggest shift was mostly observed in the case of –N=N stretching vibrations, since all of the metals were coordinated in this segment of the ligands. Besides, the spectra of 5 and 6 with perchlorate counter anions revealed a new strong peak at 1095 and 1088 cm^−1^, respectively.^42^ Likewise, new peaks identified in the FT-IR spectra of compound 8 and 11 around 1350 cm^−1^ can be assigned to the NO_3_^−^ counter anion.^43^

Fig. S1† shows the diffuse reflectance spectroscopic comparison of LM and complexes 5–11. Bands in the UV region (∼200–350 nm) can be assigned to the intra-ligand transitions such as π–π* and n–π*. Moreover, complex 5, exhibits a weak band at *λ* = 570 nm. This latter one corresponds to a d–d transition as earlier observed at *λ* = 589 nm for the nickel complex [Ni(dpbmp)_2_](ClO_4_)_2_ with dpbmp = diethyl-1,1′-(pyridine-2,6-diyl)bis(5-methyl-1*H*-pyrazole-3-carboxylate).^44^ Complex 6 also displayed one band at *λ* ∼ 490 nm, associated to a d–d transition in an octahedral surrounding.^45^ On the other hand, copper complex 8 also exhibited a large band around 730 nm, that can be ascribed to a d–d transition, aligning with the distorted octahedral structure usually observed in Cu(ii) complexes.^46^ The cadmium 7 and the zinc 10, 11 complexes do not adsorb in the visible range.^47,48^

### 3.3. Single crystals X-ray analysis

**Fig. 2:**
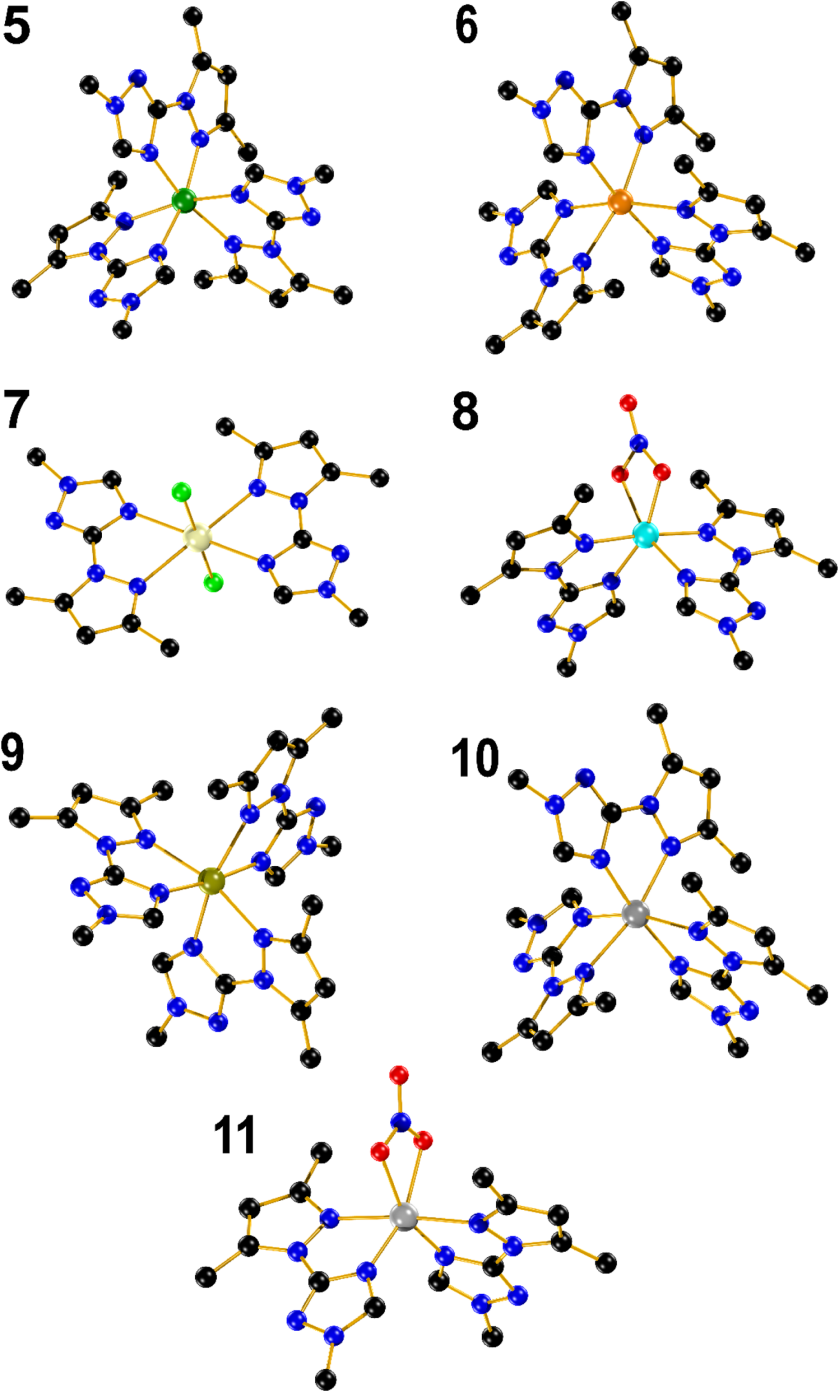
Ball and stick representation of complexes 5–11. Colour code: Ni, dark green; Co, orange; Cd, light yellow; Cu, cyan; Fe, dark yellow; Zn, grey. Hydrogen atoms and counterions were omitted for clarity.

Compound 5 crystallizes in the triclinic system, space group space *P*1̄ (#2). The crystal structure shows a nickel mononuclear complex coordinated with three LM ligand through their nitrogen atoms (Fig. 2). The coordination geometry is distorted octahedral, with bond angles [∠N–Ni–N = 77.18(9)–100.49(9)°] containing six nitrogen atoms of three bidentate LM ligands. The bond lengths between the Ni(ii) and the nitrogen from the pyrazole rings, range from 2.136(2) to 2.142(2) Å. The bond lengths between the Ni(ii) and the nitrogen from the triazole ring was found to be shorter and ranges between 2.072(2) and 2.080(2) Å. Two distorted perchlorate counter anions with a DMSO solvent molecule are also present in the asymmetric unit.

Compound 6 also crystallizes in the triclinic system, space group *P*1̄ (#2). The Co(ii) occupy the centre of a distorted octahedral coordination sphere [∠N–Co–N = 75.84(8)–101.25(8)°], which is completed by three triazolic nitrogen and three pyrazolic nitrogen atoms provided by the three branches of LM. Moreover, the N_t_–Co triazole bonds (2.100(2)–2.125(2) Å) are also slightly shorter when compared to N_p_–Co pyrazole bonds (2.170(2)–2.173(2) Å). Two perchlorate counter anions identified in the unit cell, in addition to a distorted diethyl ether can be spotted in the crystal lattice. Compound 9 adopts a crystalline structure within the triclinic system, in *P*1̄ space group. In a similar fashion to 5 and 6, the structure reveals a distorted octahedral complex with angles falling within the range [∠N–Fe–N = 74.03(8)–102.94(8)°], while two distorted non coordinated tetrafluoroborate anions are present in the crystal lattice. The bond length between Fe^II^ and nitrogen atoms is relatively long ranging from 2.152(2) to 2.216(2) Å, which fall in the range of expected values for a high-spin (HS) state for Fe^II^ ions.^49^ It is almost equal to the Fe–N bond lengths found for the polymeric 2D material, [Fe(btre)_2_(NCS)_2_] (btre = 1,2-bis(1,2,4-triazol-4-yl)ethane), which is known to not be switchable neither by temperature, pressure or red light irradiation.^50^ Such long distance observed in 9 thus hardly precluded a HS to low-spin transition to occur. A magnetic measurement performed over the range (300–5 K) confirmed a full HS state (Fig. S2†). A Mössbauer spectrum recorded at 298 K on 9 confirmed the HS state of Fe(ii), with an isomer shift *δ* = 1.056(1) mm s^−1^ and a quadrupole splitting Δ*E*_Q_ = 1.34(2) mm s^−1^. The second signal with *δ* = 0.24(6) mm s^−1^ and a quadrupole splitting Δ*E*_Q_ = 0.54(1) mm s^−1^ was ascribed to sample oxidation due to the long acquisition time in air (Fig. S2†). Complex 10 is isostructural to complexes 6 and 9, with angles spanning the range [∠N–Zn–N = 75.16(7)–101.03(7)°]. The N_t_–Zn and N_p_–Zn bond lengths fall within the range of 2.110(18)–2.151(19) Å, and 2.191(2) to 2.211(2) Å, respectively.

Complex 7 is a mononuclear cadmium complex which crystallizes in the monoclinic system, *P* 2_1_/*c* space group. The coordination geometry around Cd(ii) is a slightly distorted octahedral arrangement, with bond angles of [∠N–Cd–N = 89.24(6)–90.76(8)°]. Additionally, the distances between Cd and the nitrogen atoms in the two triazole rings are equal (2.411(3) Å) each, and the same applies to the N–Cd bond lengths with the two pyrazole rings, with both 2.414(2) Å. In a comparable manner to [CdL_2_Cl_2_] (3), 7 is coordinated to two bidentate LM ligands in the equatorial positions and two chloride anions in the axial positions with the same Cd–Cl distance of 2.543(9) Å and the Cl–Cd–Cl bond angle is perfectly linear (180.0°).

Complex 8, crystallizes in the monoclinic system, *C* 2/*c* space group. The structure shows a mononuclear copper complex coordinated with two bidentate LM ligands. The Cu(ii) coordination sphere adopts a distorted octahedral arrangement, characterized by bond angles measuring [∠N–Cu–N = 79.22(9)–104.36(13)°], involving four nitrogen's from the two bidentate LM ligands, as well as two oxygen from the coordinated nitrate counter anion in apical position. The bond distances between the metal and oxygen, triazolic and pyrazolic nitrogen atoms are 2.018(5), 2.112(2) and 2.042(2) Å, respectively, which contrasts to the trend observed with the rest of complexes where a longer N_t_–Cu bond length was observed with the triazolic nitrogen when compared to the N_p_–Cu bond length with the pyrazolic one. Compound 11, also crystallizes in the *C* 2/*c* space group, with a mononuclear Zn(ii) complex coordinated in a octahedral fashion to four nitrogen atoms from two bidentate chelating LM ligands and two oxygen atoms from nitrate counter anions [∠N–Zn–N = 77.57(5)–105.08(8)°]. Furthermore, the bond distances between Zn(ii) and oxygen, triazole and pyrazole nitrogen atoms are 2.168(14), 2.063(14) and 2.188(14) Å, respectively.

### 3.4. Cytotoxicity against MDA-MB-468

**Fig. 3:**
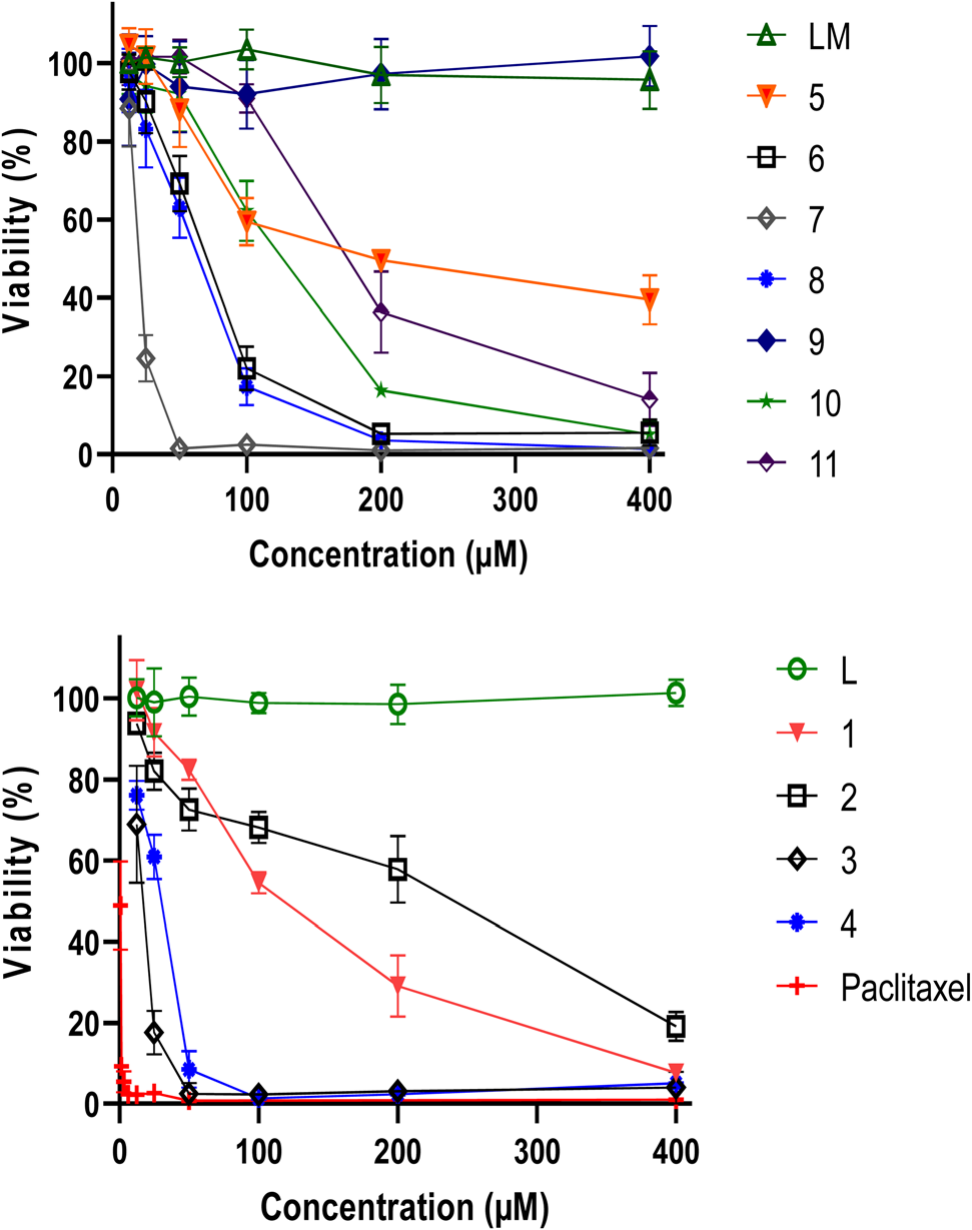
Viability of MDA-MB-486 tumor cells following 48 h treatment with different concentrations of L, LM and their metal complexes 1–11, evaluated by MTT assay. Results are means ± SD from three independent experiments. Paclitaxel was used as reference compound.

The viability of MDA-MB-486 tumour cells was assessed using the MTT assay after a 48 h treatment with varying concentrations of L, LM and their metal complexes 1–11. Both ligands L and LM did not exhibit any effect, in contrast to our metal complexes, in particular 3, 4 and 7 (Fig. 3).

Such enhanced cytotoxicity suggests that coordination of the ligands with specific metal ions resulted in a synergistic effect that amplified their anticancer properties, facilitating their interactions with cellular components and promoting cellular uptake. Interestingly, complexes 3 and 7 demonstrated remarkable cytotoxicity against tumor cells, with complex 4 showing also superior characteristics. At concentrations below 60 μM, the cadmium complexes completely destroyed the cells, indicating a potent anticancer effect.

**Fig. 4:**
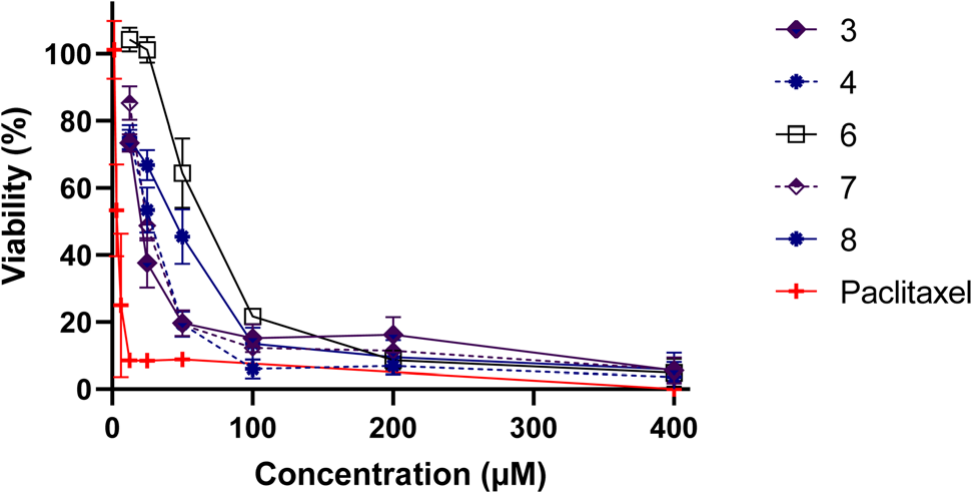
Viability of PBMC cells following 48 h treatment with different concentrations of L, LM and their metal complexes, evaluated by MTT assay. Results are means ± SD from three independent experiments. Paclitaxel was used as a reference material.

In order to validate these findings, it is essential to assess the cytotoxic activity of the metal complexes on non-cancer cells, which could help determine their safety and selectivity index (Fig. 4). Table 1 highlights the inhibitory concentration 50 (IC_50_) in μM and selectivity index of L, LM ligands and their metal complexes against MDA-MB-486 cancer cell line and normal PBMCs cells. Whereas 4 displayed a selectivity index of 0.97, indicating a lack of reliability as a TNBC antitumor drug, 3 and 7, presented selectivity index values of 1.34 and 1.30, respectively. These results suggest that 3 and 7 hold greater potential as TNBC anticancer agents, with outstanding IC_50_ values of 15.61 and 19.90, respectively, against the MDA-MB-486 cancer cell line. Remarkably, 3 and 7 are both made of only two ligands, leaving two positions occupied by independent chlorine atoms, which are made available to better interact with cellular components, leading to such beneficial properties.

**Table 1:** Inhibitory concentration 50 (IC_50_) in μM and selectivity index of L, LM ligands and their metal complexes against MDA-MB-486 cancer cell line and normal PBMCs cells^a^

Molecules	IC_50_ (μM)		Selectivity index^b^
	MDA-MB-468	PBMCs	
L	NA	—	—
1	114.3 ± 5.22^c^	—	—
2	178 ± 31.48^e^	—	—
3	15.61 ± 2.18^d^	20.97 ± 2.67^c^	1.34
4	25.85 ± 2.43^fd^	24.97 ± 3.07^c^	0.97
LM	NA	—	—
5	215.8 ± 19.95^a^	—	—
6	64.60 ± 5.72^b^	64.18 ± 5.95^b^	0.99
7	19.90 ± 2.79^d^	25.96 ± 1.25^c^	1.30
8	56.84 ± 7.4^b^	37.43 ± 5.88^a^	0.66
9	NA	—	—
10	116.7 ± 10.38^c^	—	—
11	182.6 ± 20.26^ae^	—	—
Paclitaxel	0.74 ± 0.12^d^	3.92 ± 0.96^d^	5.31

a Each value represents the average of three separate experiments. Distinct letters denote significant differences between the treatments (*p* < 0.05).

b IC_50_ (PBMCs)/IC_50_ (MDA-MB-468).

## 4. Conclusion

In conclusion, the reaction of LM, with assorted metal salts led to the formation of a family of seven novel coordination complexes with diverse coordination environments. This further confirms the versatility of 1,2,4-triazole derivatives in coordination chemistry. Most importantly, the evaluation of MDA-MB-486 tumor cell viability following treatment with varying concentrations of ligands L, LM, and their metal complexes revealed intriguing findings. The coordination complexes exhibited significantly improved performance compared to their respective ligands, indicating that the ligand–metal coordination has a profound effect on the cytotoxic activity of the resulting complexes. Although cadmium alone has shown to promote the proliferation of TNBC in numerous reported studies,^51,52^ the newly emerged cadmium complexes 3 and 7 not only demonstrated effective inhibition against MDA-MB-468 cell lines but also showcased a selectivity index exceeding 1. This conclusively demonstrates a contrary effect to proliferation. Moreover, it is important to note that exposure to cadmium can result in diverse impacts on human health, including kidney damage, cardiovascular effects, and bone damage. Consequently, it will be necessary to conduct thorough investigations into the safety and potential drawbacks of these novel compounds. Despite the significant gap in the literature regarding the development of efficient coordination materials for triple negative breast cancer, which is known to be a challenging form of tumor cells, this pioneering study paves the way for exciting opportunities and avenues to explore. Further investigations into the underlying mechanisms of action and *in vivo* studies are warranted to fully understand the therapeutic potential and safety profile of these coordination complexes for cancer treatment, as well as the syntheses of new complexes.

## Author contributions

YD performed synthesis, characterization and wrote the paper with YG. SR and YG both designed and managed the project. YB contributed to the synthesis of coordination complexes. AI and AZ performed antitumor experiments. AEM contributed with organic synthesis. HNM and MF performed X-ray studies. AR performed SQUID characterization.

## Conflicts of interest

There are no conflicts to declare.

## Supplementary Material

RA-013-D3RA07714D-s001

RA-013-D3RA07714D-s002
