# Supplementary material for: New triazole-based coordination complexes as antitumor agents against triple negative breast cancer MDA-MB-468 cell line
Source: RSC Adv. 2023 Dec 12;13(51):36158–67. doi: 10.1039/d3ra07714d (PMC10714199; doi:10.1039/d3ra07714d)
Supplement: RA-013-D3RA07714D-s001 [file RA-013-D3RA07714D-s001.pdf]

Electronic Supplementary Material (ESI) for RSC Advances.
This journal is © The Royal Society of Chemistry 2023

Electronic Supplementary document (ESI)

New Triazole-based Coordination Complexes as Antitumor Agents against Triple Negative Breast Cancer MDA-MB-468 Cell Line

Youssef Draoui,[1] Smaail Radi,[*,1] Yousra Bahjou,[1,2] Abderrazaka Idir,[3] Amal El Mahdaoui,[1] Abdelmajidb Zyad,[3] Haralampos N. Miras,[4] Marilena Ferbinteanu,[5] Aurelian Rotaru,[6] Yann Garcia[*,2]

[1] *LCAE, Department of Chemistry, Faculty of Science, University Mohamed I , P.O. Box 524, Oujda 60 000, Morocco.*

[2] *Institute of Condensed Matter and Nanosciences, Molecular Chemistry, Materials and Catalysis (IMCN/MOST), Université catholique de Louvain, Place L. Pasteur 1, 1348 Louvain-la-Neuve, Belgium.*

[3] *Team of Experimental Oncology and Natural Substances, Cellular and Molecular Immuno pharmacology, Faculty of Science and Technology, Sultan Moulay Slimane University, Beni-Mellal, Morocco.*

[4] *School of Chemistry, Joseph Black Building, University of Glasgow, Glasgow G12 8QQ, UK.*

[5] *Inorganic Chemistry Department, Faculty of Chemistry, University of Bucharest, Panduri Road, no. 90, Bucharest 050663, Romania*

[6] *Department of Electrical Engineering and Computer Science & Research Center MANSiD, "Stefan cel Mare" University, University Street, No. 13, Suceava 720229, Romania*

E-mail: yann.garcia@uclouvain.be ; s.radi@ump.ac.ma

Content:

Table S1. Summary of crystallographic data and refinement for **5-11**……………………………………………………………3

Table S2. Selected bond lengths [Å] and angles [°] for **5-11**……………………………………………………………………..4

^{1}H NMR LM………..……5

^{13}C NMR LM………..6

Figure S1. Diffuse reflectance spectroscopic comparison of **LM** and **5-11**………………………………………………………7

Figure S2. SQUID measurement on **9** carried out over the temperature range (300-5 K) over three consecutive runs……….…..8

Figure S3. Mössbauer spectrum in transmission mode of **9** recorded at room temperature…………………………….………..9

HRMS of LM……….10

HRMS of 5…….11

HRMS of 6…….12

HRMS of 7…….13

HRMS of 8…….14

HRMS of 10……………………………………………………………………………………………..…………….15

Table S1. Summary of crystallographic data and refinement for **5-11**.

Compounds	(**5**)	(**6**)	(**7**)	(**8**)	(**9**)	(**10**)	(**11**)
Empirical formula	$C_{26}H_{39}Cl_2N_{15}NiOS$	$C_{52}H_{76}Cl_4Co_2N_{30}O_{17}$	$C_{16}H_{22}CdCl_2N_{10}$	$C_{16}H_{22}CuN_{12}O_6$	$C_{24}H_{33}B_2F_8FeN_{15}$	$C_{24}H_{33}B_2F_8ZnN_{15}$	$C_{16}H_{22}N_{12}ZnO_6$
Formula weight	867.39	1653.08	537.73	541.99	761.12	770.64	543.82
Crystal size (mm)	0.30 x 0.20 x 0.10	0.30 x 0.14 x 0.10	0.60 x 0.40 x 0.30	0.50 x 0.30 x 0.25	0.40 x 0.35 x 0.15	0.50 x 0.47 x 0.20	0.20 x 0.18 x 0.15
Crystal system	Triclinic	triclinic	monoclinic	Monoclinic	triclinic	Triclinic	monoclinic
Space group	*P -1* (#2)	*P -1* (#2)	*P* 2_1/*c* (#14)	*C* 2/*c* (#15)	*P -1* (#2)	*P -1* (#2)	*C* 2/*c* (#15)
a (Å)	11.1956(13)	11.7384(12)	7.1207(3)	15.2108(11)	11.6977(19)	11.7208(8)	15.3500(5)
b (Å)	14.8446(14)	11.7537(11)	17.0820(8)	14.0236(9)	11.7249(9)	11.7347(10)	13.6196(4)
c (Å)	14.8844(18)	15.3103(9)	8.8551(4)	11.5087(9)	15.229(2)	15.1727(9)	11.4186(3)
α (°)	119.056(11)	80.114(7)	90	90	79.371(9)	79.553(6)	90
β (°)	92.646(10)	78.156(8)	94.076(2)	108.678(9)	79.491(12)	79.334(6)	109.496(3)
γ (°)	110.233(10)	73.278(9)	90	90	73.371(10)	73.196(7)	90
Volume ($Å^3$)	1958.6(4)	1965.6(3)	1074.37(8)	2325.6(3)	1948.3(5)	1945.0(3)	2250.31(12)
Z	2	1	2	4	2	2	4
ρcalc.(g/cm^3)	1.471	1.397	1.662	1.548	1.297	1.316	1.605
F(000)	900	856	540.00	1116	780	788	1120
μMoK_α (cm^{-1})	0.752	0.637	1.291	0.999	0.463	0.707	1.153
T (K)	293(2)	293(2)	293	293(2)	293(2)	297(2)	150(2)
θ min/max	3.157/26.158	3.035/26.203	3.106/ 27.500	3.455/26.040	2.873/26.165	3.096/26.157	2.454/53.701
Refl. collected/unique	30651/7776	27103/7827	4681/2316	7610/2272	20213/ 7758	15416/7190	17389/7841
Data/restraints/parameters	7776/156/ 555	7827/50/551	2316/0/133	2272/62/194	7758/60/522	7190/110/506	7841/1/178
Goodness of fit on F^2	1.046	1.068	1.065	1.078	1.068	1.030	1.056
Final *R* indices [I>2σ(I)]	R_1= 0.0514, wR_2=0.1412	R_1=0.0478, wR_2= 0.1397	R_1 = 0.0469, wR_2 = 0.1278	R_1=0.0452, wR_2=0.1284	R_1=0.0554, wR_2=0.1499	R_1=0.0456, wR_2=0.1268	R_1=0.0587, wR_2=0.1700
R indices (all data)	R_1= 0.0584, wR_2=0.1462	R_1= 0.0558, wR_2=0.1458	R_1 = 0.0489, wR_2 = 0.1312	R_1= 0.0472, wR_2=0.1299	R_1=0.0723, wR_2=0.1635	R_1=0.0506, wR_2=0.1304	R_1=0.0990, wR_2=0.1894

Table S2. Selected bond lengths [Å] and angles [°] for **5-11.**

Compound	Bond lengths, Å		Bond angles, °	
**5**	Ni(1)-N(30)	2.072(2)	N(30)-Ni(1)-N(2)	91.94(9)
	Ni(1)-N(10)	2.073(2)	N(30)-Ni(1)-N(22)	77.68(8)
	Ni(1)-N(50)	2.080(2)	N(50)-Ni(1)-N(22)	93.84(9)
	Ni(1)-N(2)	2.136(2)	N(2)-Ni(1)-N(22)	98.81(8)
	Ni(1)-N(22)	2.138(2)	N(50)-Ni(1)-N(42)	77.18(9)
	Ni(1)-N(42)	2.142(2)	N(2)-Ni(1)-N(42)	100.49(9)
**6**	Co(1)-N(10)	2.100(2)	N(10)-Co(1)-N(2)	77.14(8)
	Co(1)-N(50)	2.116(2)	N(50)-Co(1)-N(2)	95.50(8)
	Co(1)-N(30)	2.125(2)	N(30)-Co(1)-N(22)	75.84(8)
	Co(1)-N(2)	2.170(2)	N(2)-Co(1)-N(22)	89.40(9)
	Co(1)-N(22)	2.170(2)	N(10)-Co(1)-N(42)	93.70(8)
	Co(1)-N(42)	2.173(2)	N(22)-Co(1)-N(42)	101.25(8)
**7**	Cd(1)-N(3)#1	2.411(3)	N(1)#1-Cd(1)-N(1)	180.0
	Cd(1)-N(3)	2.411(3)	N(3)#1-Cd(1)-Cl(1)	89.24(8)
	Cd(1)-N(1)#1	2.414(2)	N(3)-Cd(1)-Cl(1)	90.76(8)
	Cd(1)-N(1)	2.414(2)	N(1)#1-Cd(1)-Cl(1)	89.24(6)
	Cd(1)-Cl(1)	2.5426(9)	N(3)#1-Cd(1)-Cl(1)#1	90.76(8)
	Cd(1)-Cl(1)#1	2.5426(9)	Cl(1)-Cd(1)-Cl(1)#1	180.0
**8**	Cu(1)-O(23)	2.018(5)	O(23)-Cu(1)-N(2)	86.42(17)
	Cu(1)-N(2)	2.042(2)	N(2)#1-Cu(1)-N(10)#1	79.22(9)
	Cu(1)-N(2)#1	2.042(2)	O(23)-Cu(1)-N(10)	100.97(14)
	Cu(1)-N(10)#1	2.112(2)	N(2)-Cu(1)-N(10)	79.22(9)
	Cu(1)-N(10)	2.112(2)	N(2)#1-Cu(1)-N(10)	97.82(10)
			N(10)#1-Cu(1)-N(10)	104.36(13)
**9**	Fe(1)-N(30)	2.152(2)	N(10)-Fe(1)-N(50)	87.89(9)
	Fe(1)-N(10)	2.161(2)	N(30)-Fe(1)-N(42)	102.94(8)
	Fe(1)-N(50)	2.186(2)	N(50)-Fe(1)-N(42)	74.03(8)
	Fe(1)-N(42)	2.196(2)	N(30)-Fe(1)-N(22)	75.53(8)
	Fe(1)-N(22)	2.201(2)	N(42)-Fe(1)-N(22)	92.11(9)
	Fe(1)-N(2)	2.216(2)	N(30)-Fe(1)-N(2)	94.02(8)
**10**	Zn(1)-N(30)	2.1104(18)	N(10)-Zn(1)-N(22)	95.60(8)
	Zn(1)-N(10)	2.1411(19)	N(30)-Zn(1)-N(42)	101.03(7)
	Zn(1)-N(50)	2.1512(19)	N(50)-Zn(1)-N(42)	75.16(7)
	Zn(1)-N(22)	2.191(2)	N(22)-Zn(1)-N(42)	91.87(8)
	Zn(1)-N(42)	2.191(2)	N(30)-Zn(1)-N(2)	94.04(7)
	Zn(1)-N(2)	2.211(2)	N(42)-Zn(1)-N(2)	99.48(8)
**11**	N(6)-Zn(1)	2.1884(14)	N(11)-Zn(1)-N(11)#1	105.08(8)

	N(11)-Zn(1)	2.0637(14)	N(11)#1-Zn(1)-O(30)#1	97.75(5)
	O(30)-Zn(1)	2.1682(14)	O(30)#1-Zn(1)-O(30)	59.80(7)
			N(11)-Zn(1)-N(6)	77.57(5)
			O(30)#1-Zn(1)-N(6)	95.53(5)
			O(30)-Zn(1)-N(6)	89.40(5)

^{1}H NMR LM

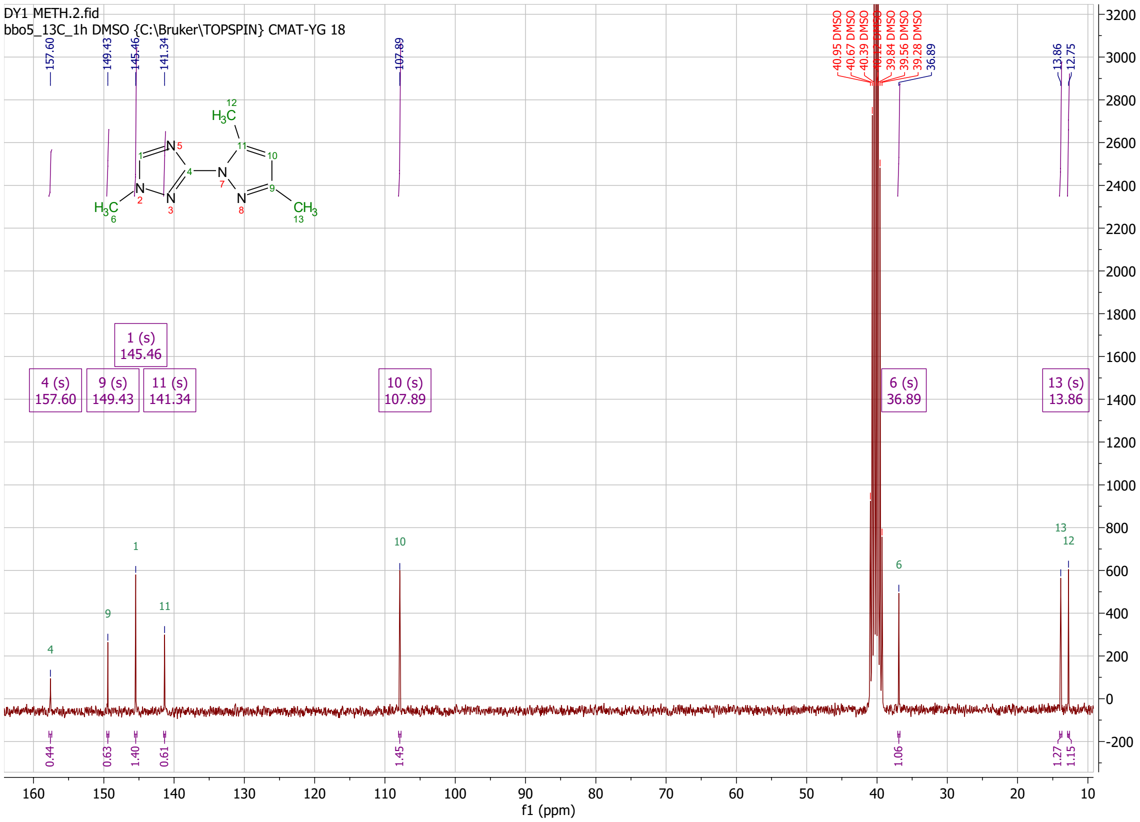

^{13}C NMR LM:

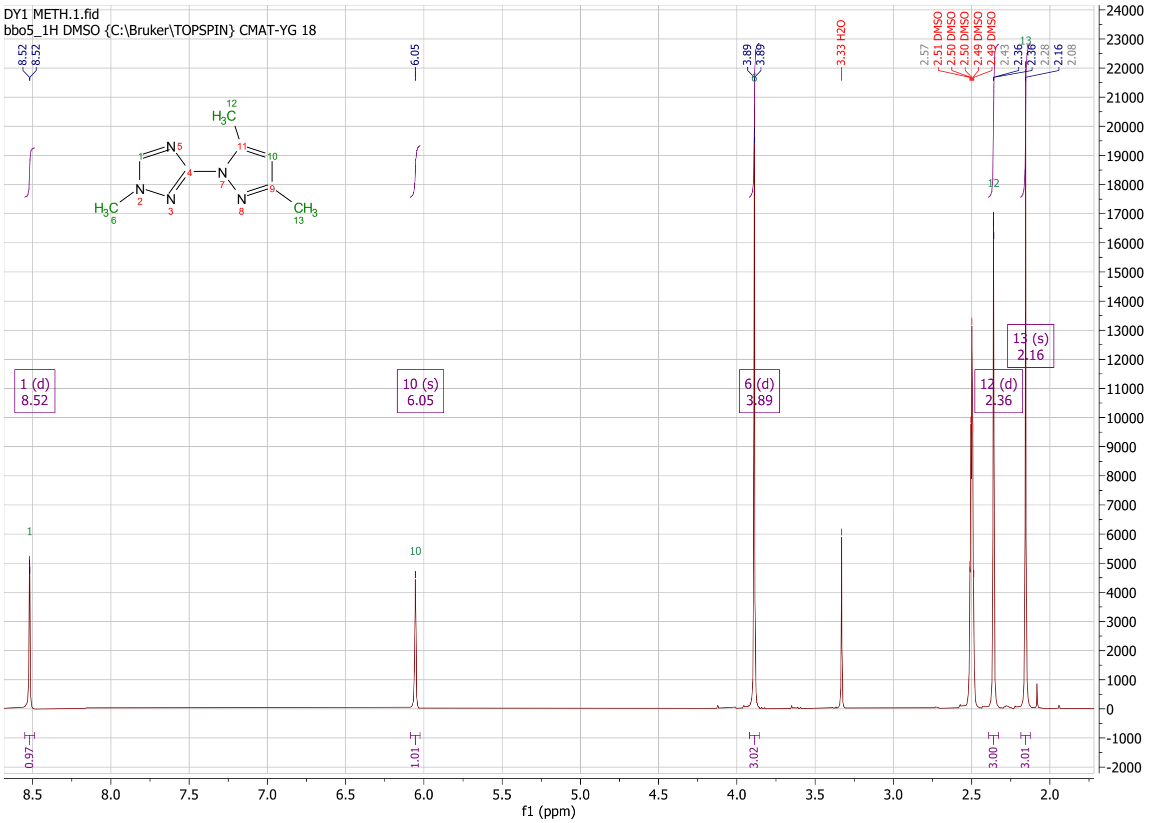
DY1 METH.1.fid
bbo5_1H DMSO {C:\Bruker\TOPSPIN} CMAT-YG 18
1 (d)
8.52
10 (s)
6.05
6 (d)
3.89
12 (d)
2.36
13 (s)
2.16
3.33 H2O
f1 (ppm)

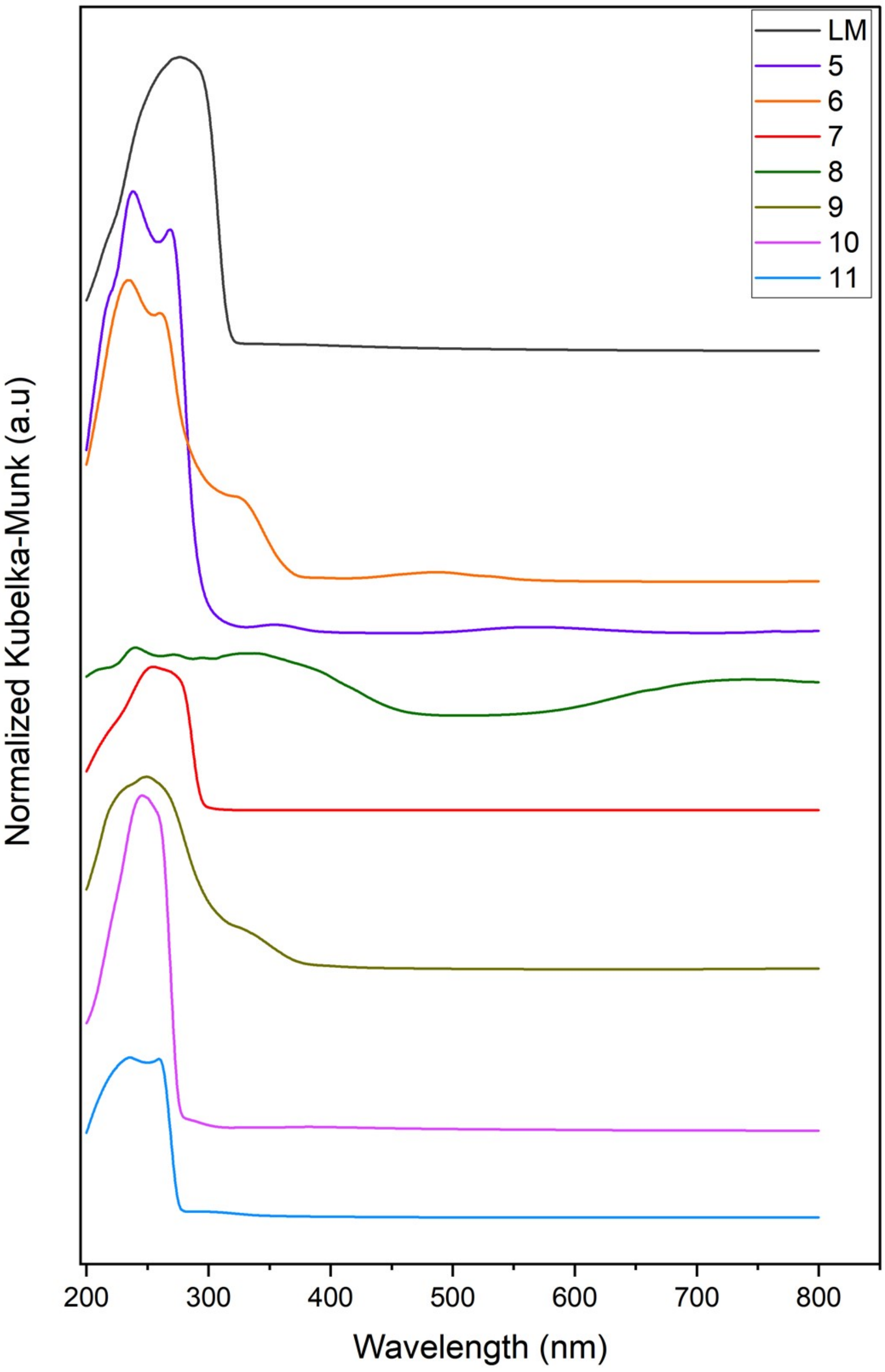

Figure S1. Diffuse reflectance spectroscopic comparison of **LM** and **5**-**11**.

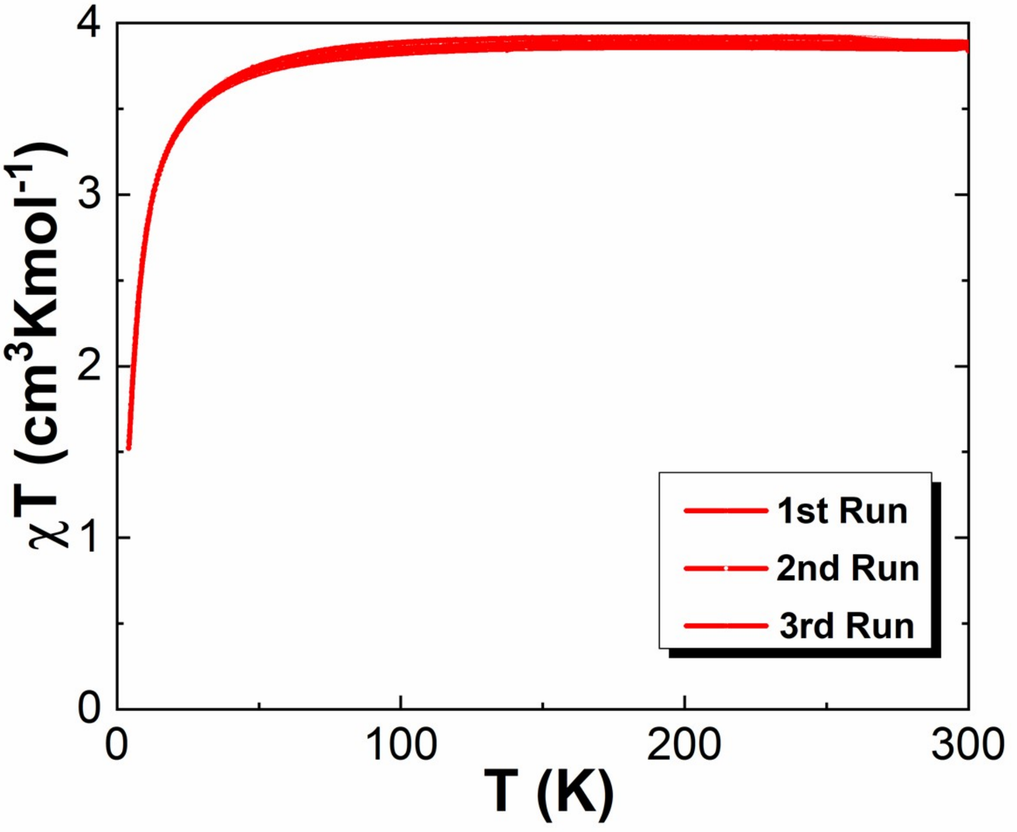

Figure S2. SQUID measurement on **9** carried out over the temperature range (300-5 K) over three consecutive runs.

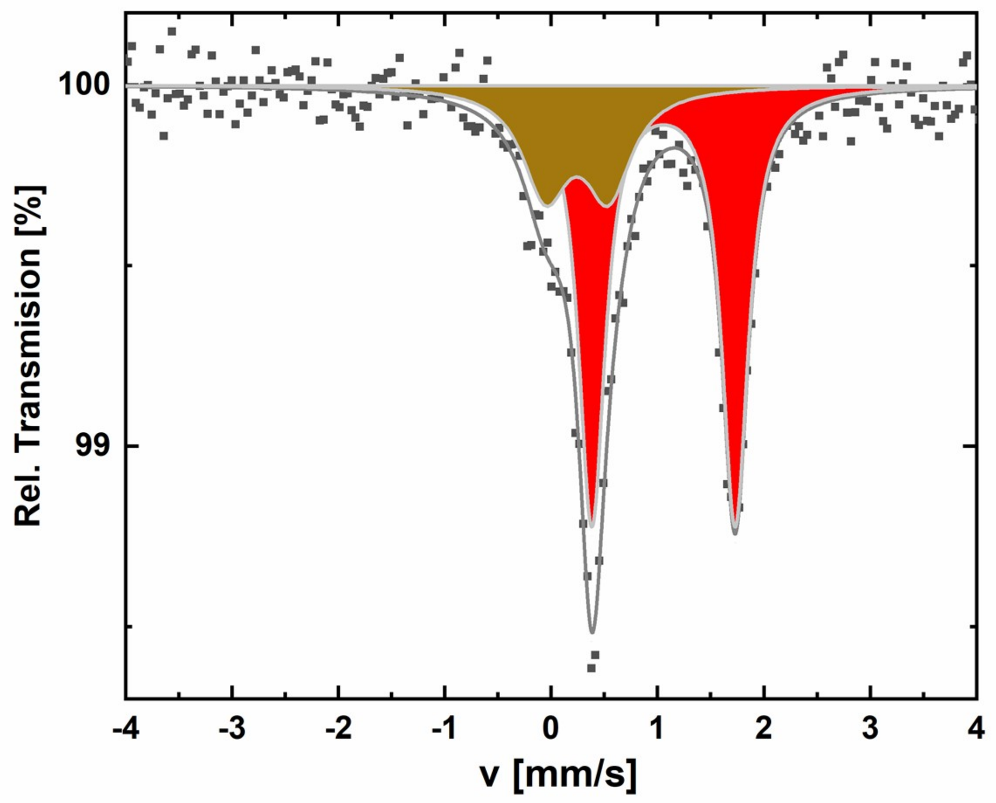

Figure S3. Mössbauer spectrum in transmission mode of **9** recorded at room temperature.

HRMS of LM:

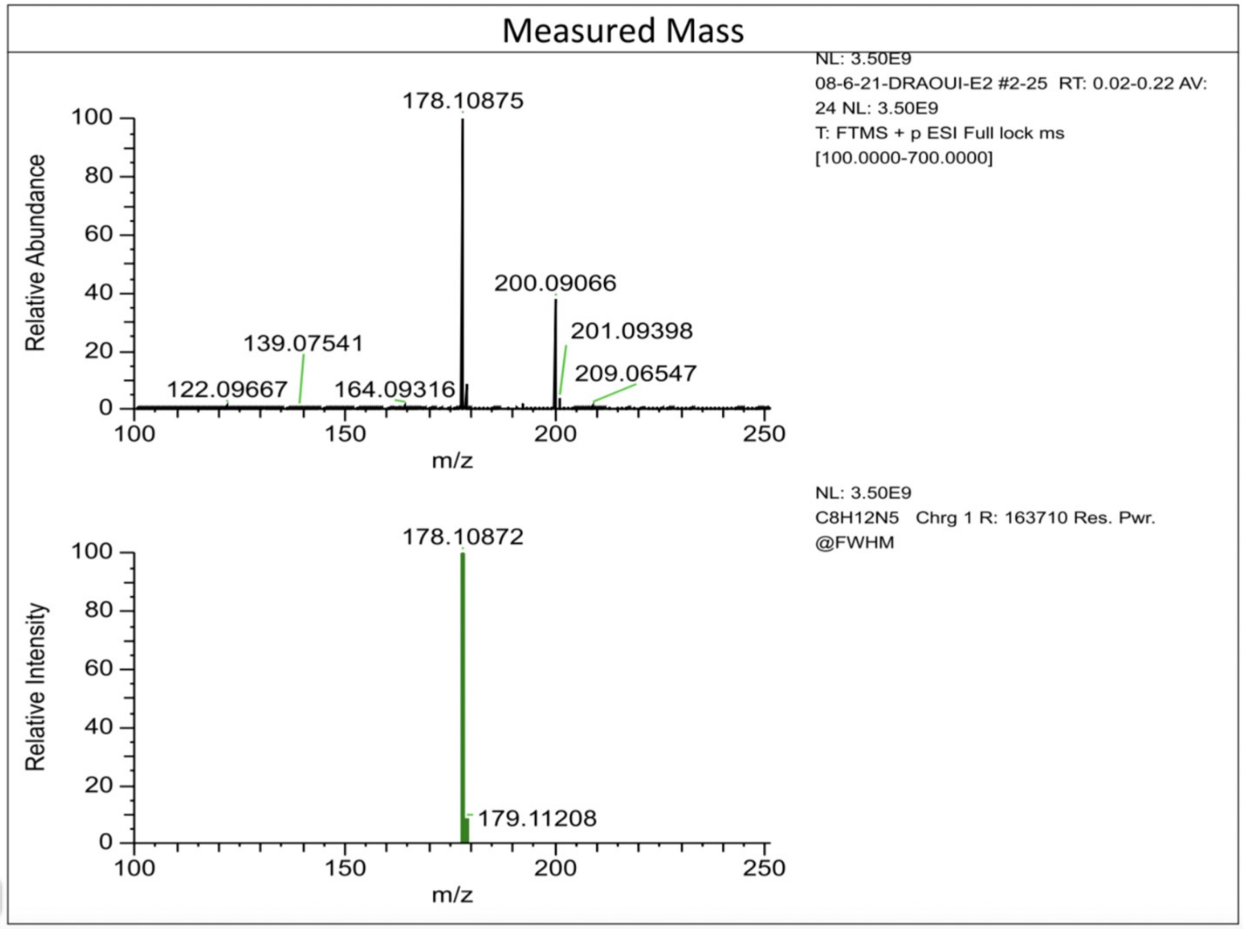

Elemental Composition Results

Peak Mass	Display Formula	MS Cov. [%]	Delta [ppm]	Theo. mass
178.10875	$C_8H_{12}N_5$	99.74	0.18	178.10872

HRMS of 5:

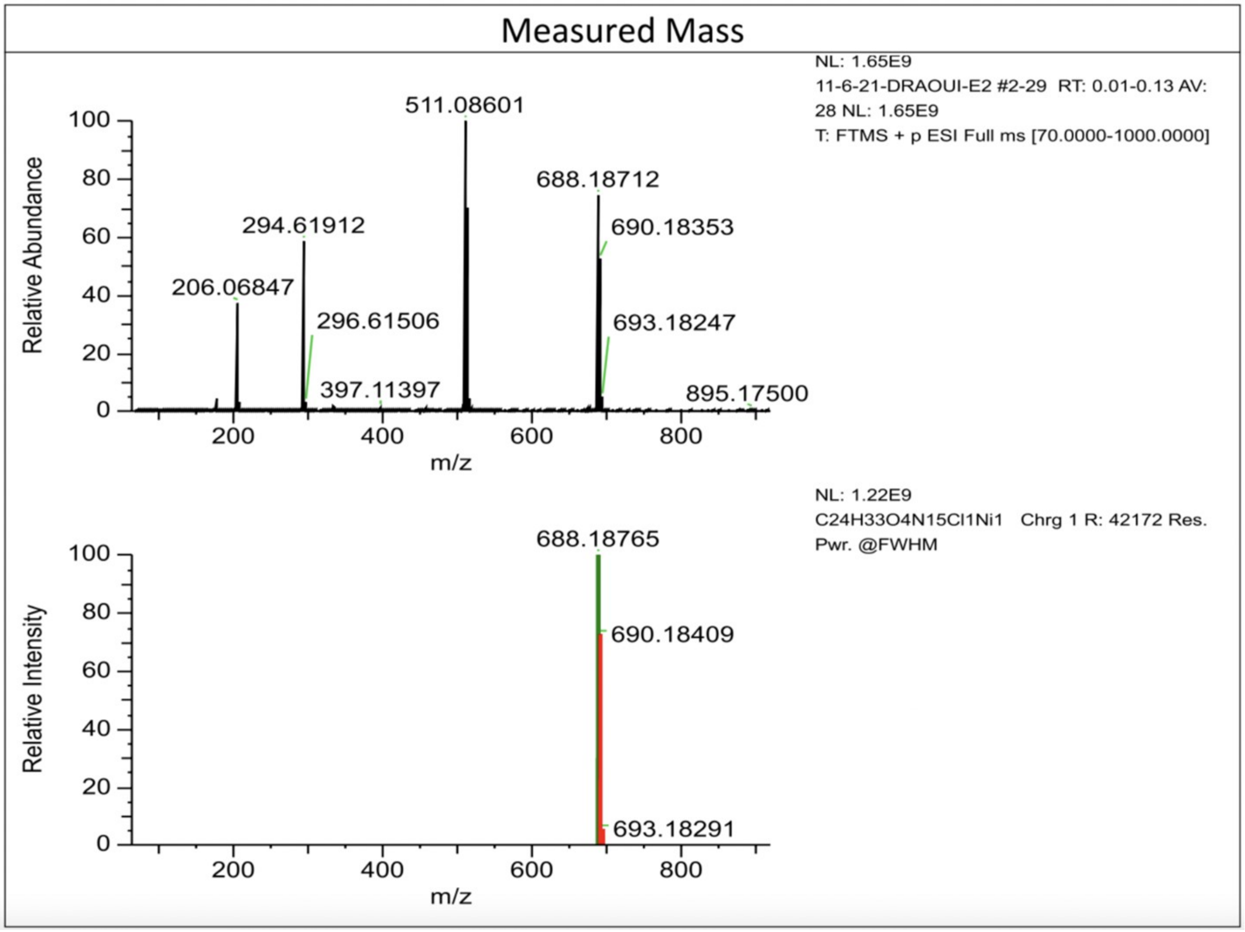

Elemental Composition Results

Peak Mass	Display Formula	MS Cov. [%]	Delta [ppm]	Theo. mass
688.18712	$C_{24}H_{33}O_4N_{15}{}^{35}Cl^{58}Ni$	99.94	-0.76	688.18765

HRMS of 6:

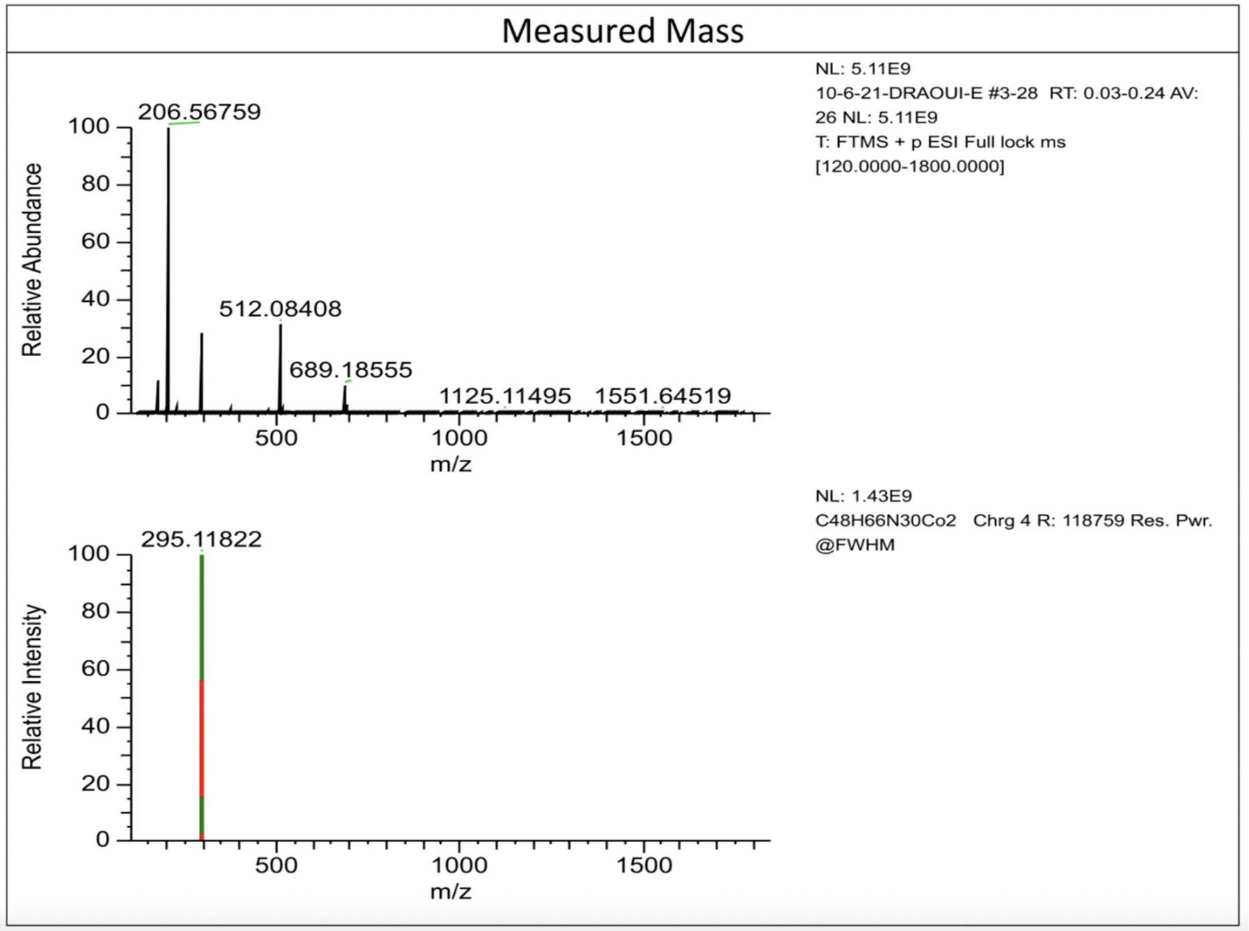

Elemental Composition Results

Peak Mass	Display Formula	MS Cov. [%]	Delta [ppm]	Theo. mass
295.11811	$C_{48}H_{66}N_{30}{}^{59}Co_2$	78.07	-0.37	295.11822

Peak Mass	Display Formula	MS Cov. [%]	Delta [ppm]	Theo. mass
295.11811	$C_{24}H_{33}N_{15}{}^{59}Co$	99.21	-0.37	295.11822

HRMS of 7:

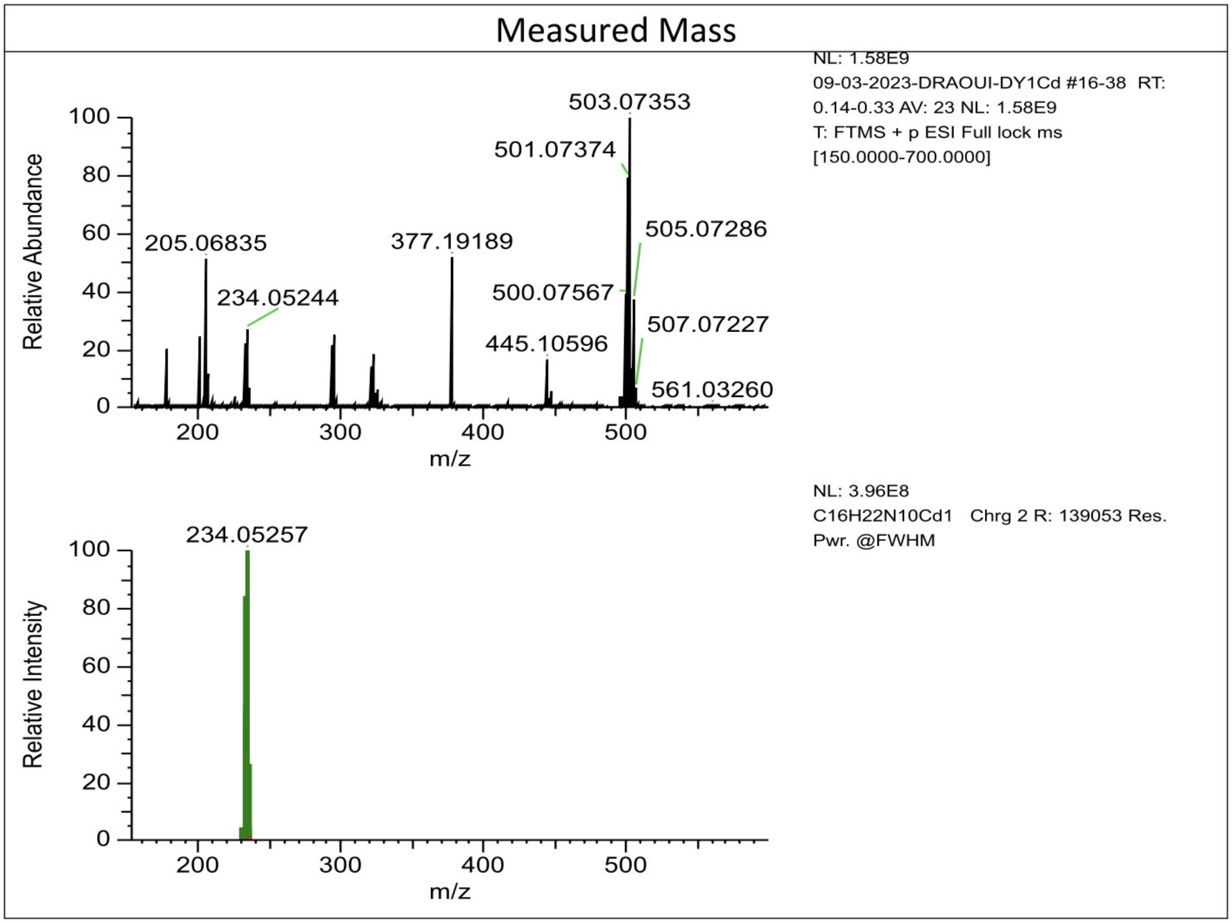

Elemental Composition Results

Peak Mass	Display Formula	MS Cov. [%]	Delta [ppm]	Theo. mass
230.05410	$C_{16}H_{22}N_{10}{}^{106}Cd$	99.53	-0.11	230.05413

HRMS of 8:

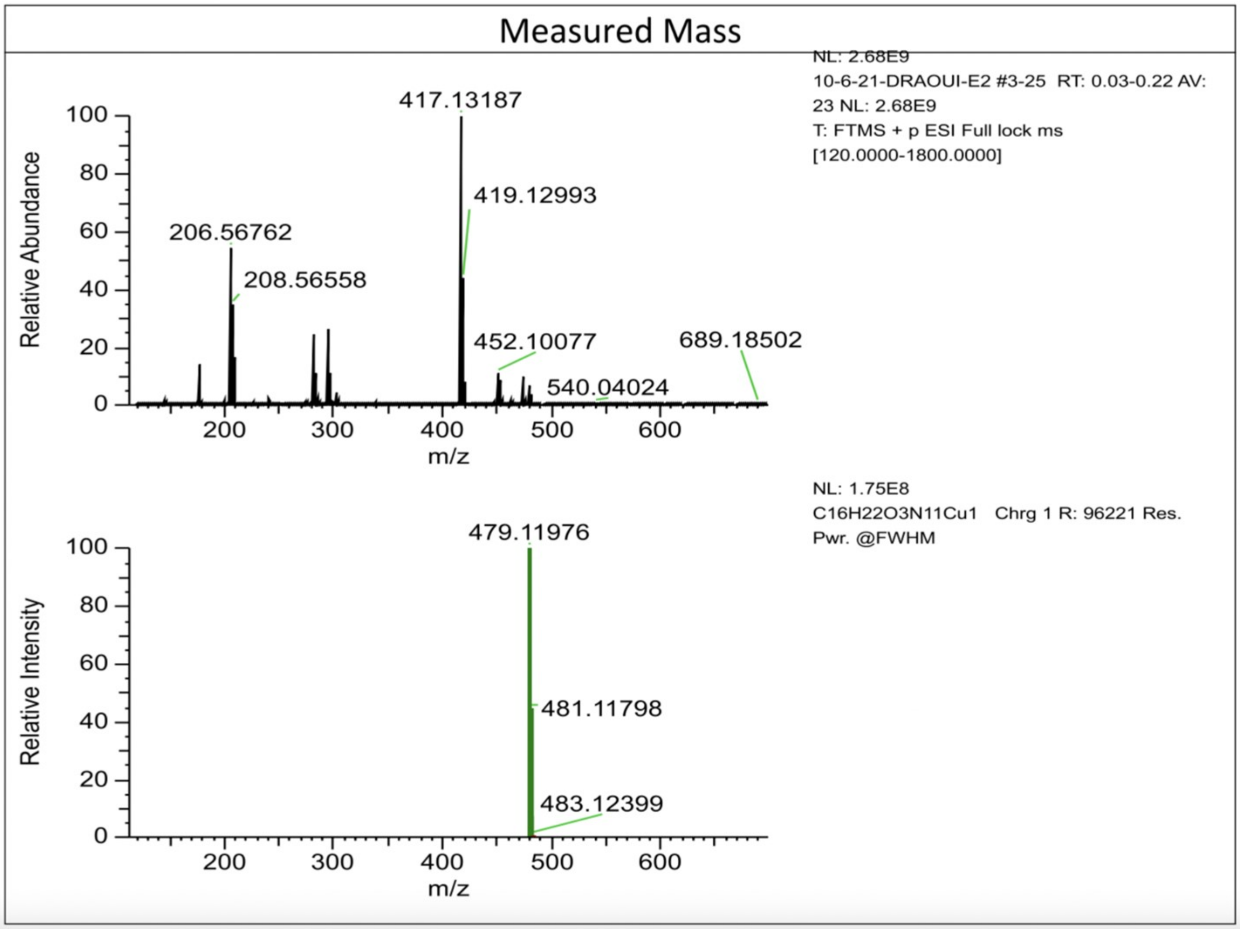

Elemental Composition Results

Peak Mass	Display Formula	MS Cov. [%]	Delta [ppm]	Theo. mass
479.11979	$C_{16}H_{22}O_3N_{11}{}^{63}Cu$	99.23	0.06	479.11976

HRMS of 10:

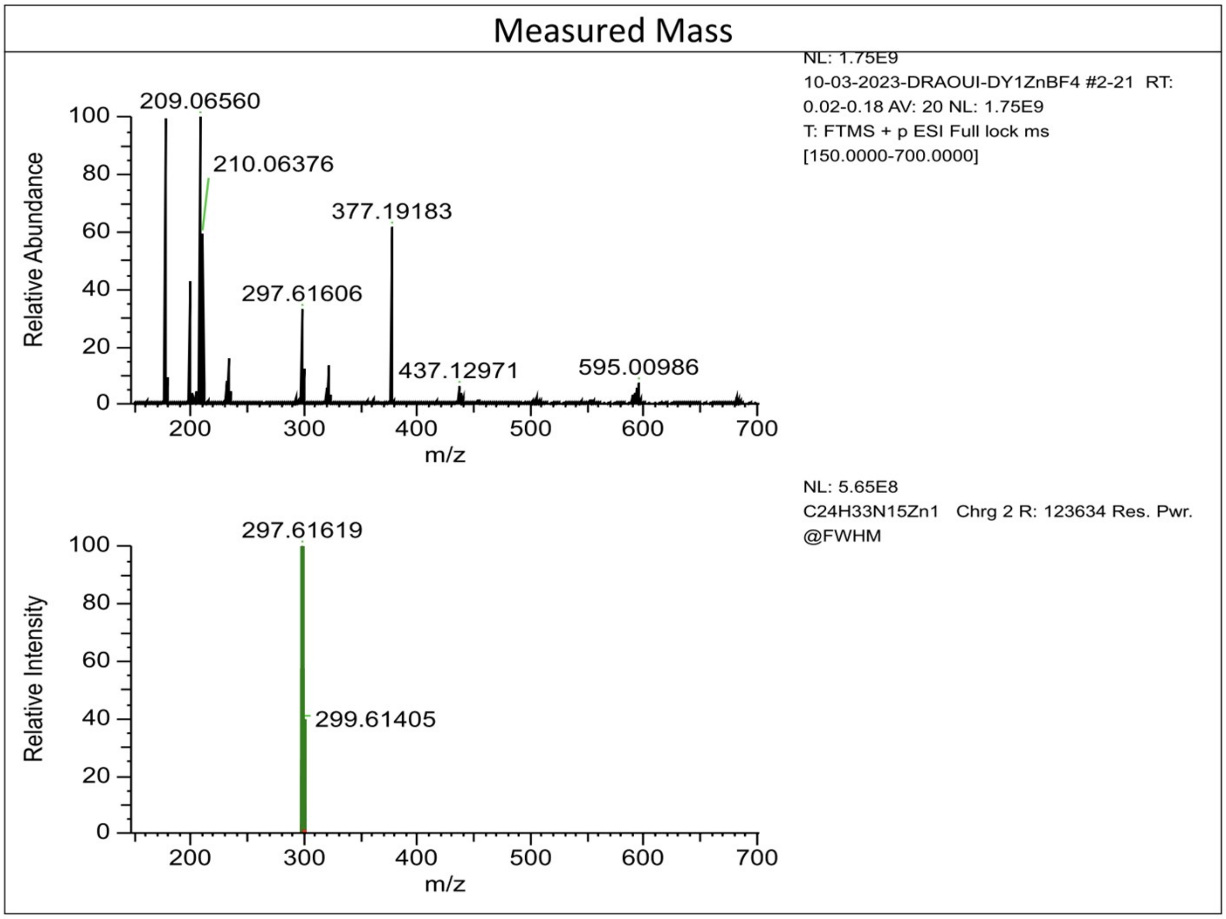

Elemental Composition Results

Peak Mass	Display Formula	MS Cov. [%]	Delta [ppm]	Theo. mass
297.61606	$C_{24}H_{33}N_{15}{}^{64}Zn$	99.02	-0.46	297.61619
